# Supplementary material for: Dissipative Particle Dynamics Simulation of the Sensitive Anchoring Behavior of Smectic Liquid Crystals at Aqueous Phase
Source: Molecules. 2022 Nov 1;27(21):7433. doi: 10.3390/molecules27217433 (PMC9658472; doi:10.3390/molecules27217433)
Supplement: Supplementary file 1 [file molecules-27-07433-s001.zip › molecules-1971836-supplementary.pdf]

Supporting Information

Dissipative Particle Dynamics Simulation of the Sensitive Anchoring Behavior of Smectic Liquid Crystals at Aqueous Phase

Shiwei Chen, Jinliang Zhang, Huilong Liu, Tongyue Qiu, Haoxiang Tang and Zunmin Zhang *

State Key Laboratory of Materials-Oriented Chemical Engineering, College of Chemical Engineering, Nanjing Tech University, Nanjing 211816, China
* Correspondence: zunmin.zhang@njtech.edu.cn

Contents

S1. Bead density distributions of representative smectic anchoring configurations

S2. Representative anchoring snapshots in an ordering process

S3. Representative anchoring snapshots in a reorientation process

S1. Bead density distributions of representative smectic anchoring configurations

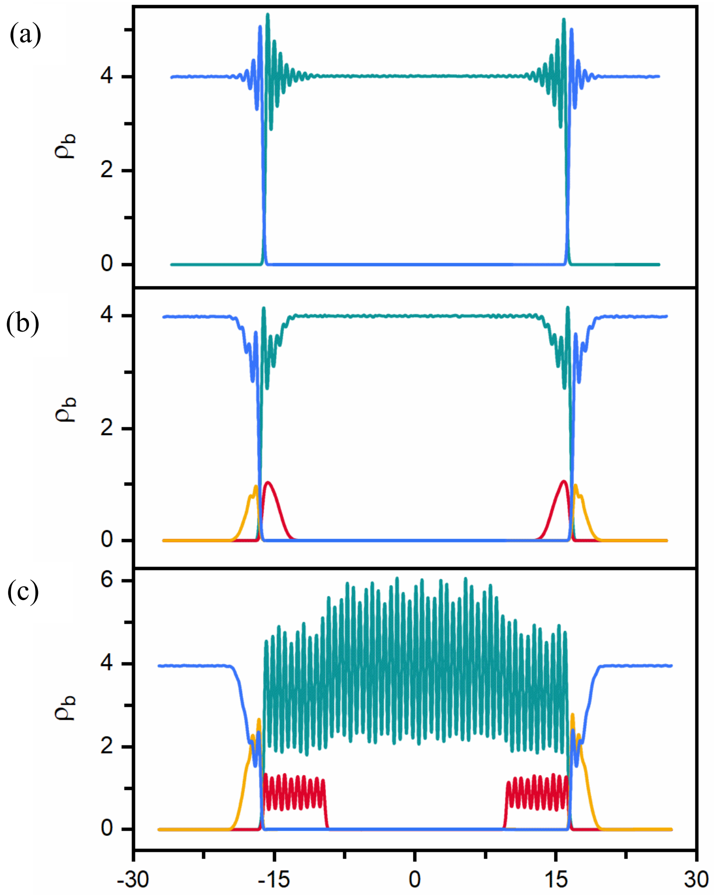

Figure S1. Bead density distributions of the mesogens (Cyan), amphiphile tails (Red), amphiphile heads (Orange) and water (Blue) in three representative anchoring configurations of smectic (T = 0.3) LCs: (a) planar anchoring at ρ_s= 0.0, (b) tilted anchoring at ρ_s = 0.2 and (c) homeotropic anchoring at ρ_s = 0.6.

S2. Representative anchoring snapshots in an ordering process

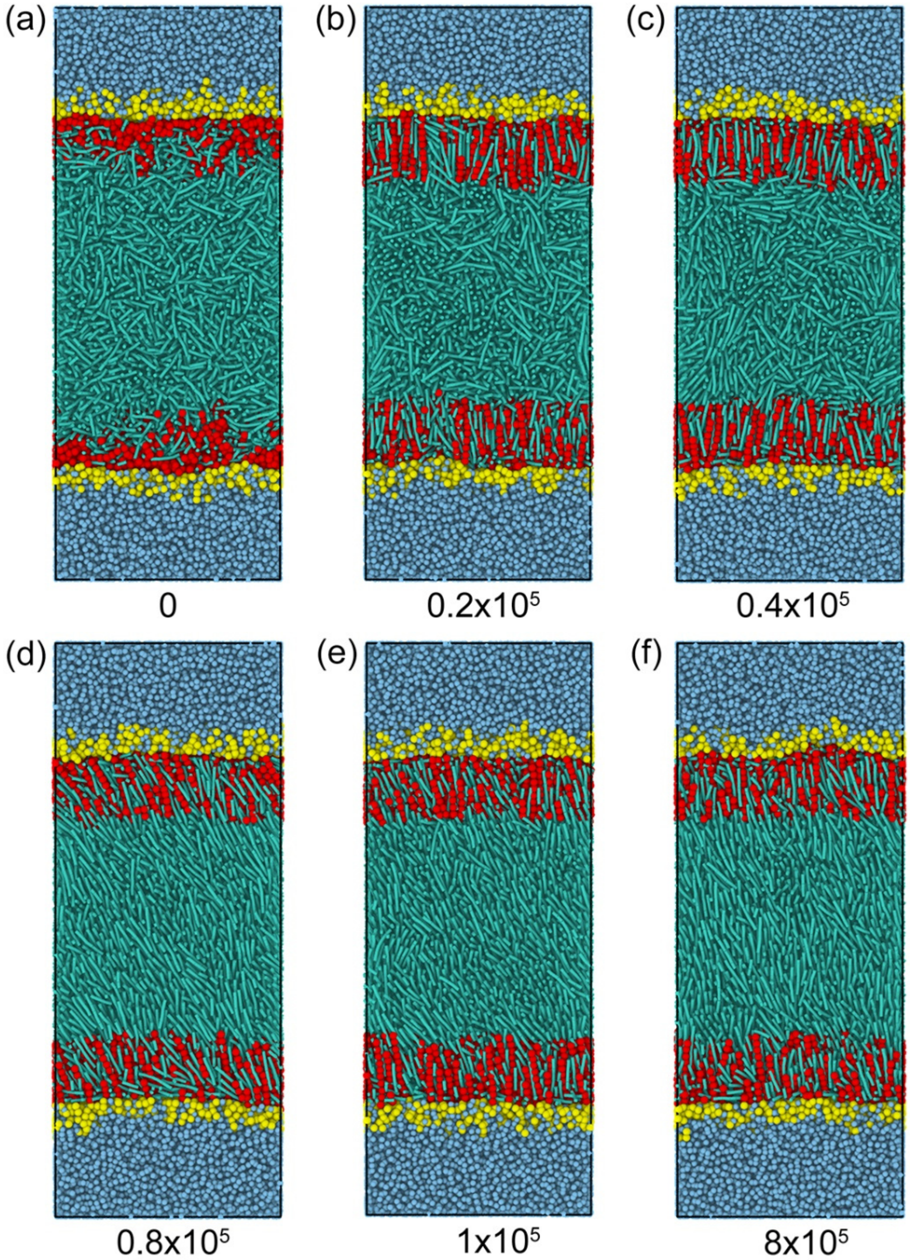

Figure S2. Instantaneous snapshots of six representative configurations in an ordering process by cooling a disordering configuration at ρ_s = 0.85 to the homeotropic nematic anchoring at T = 0.6, when (a) t = 0, (b) t = 0.2×10^5 time steps, (c) t = 0.4×10^5 time steps, (d) t = 0.8×10^5 time steps, (e) t = 1.0×10^5 time steps and (f) t = 8.0×10^5 time steps.

S3. Representative anchoring snapshots in a reorientation process

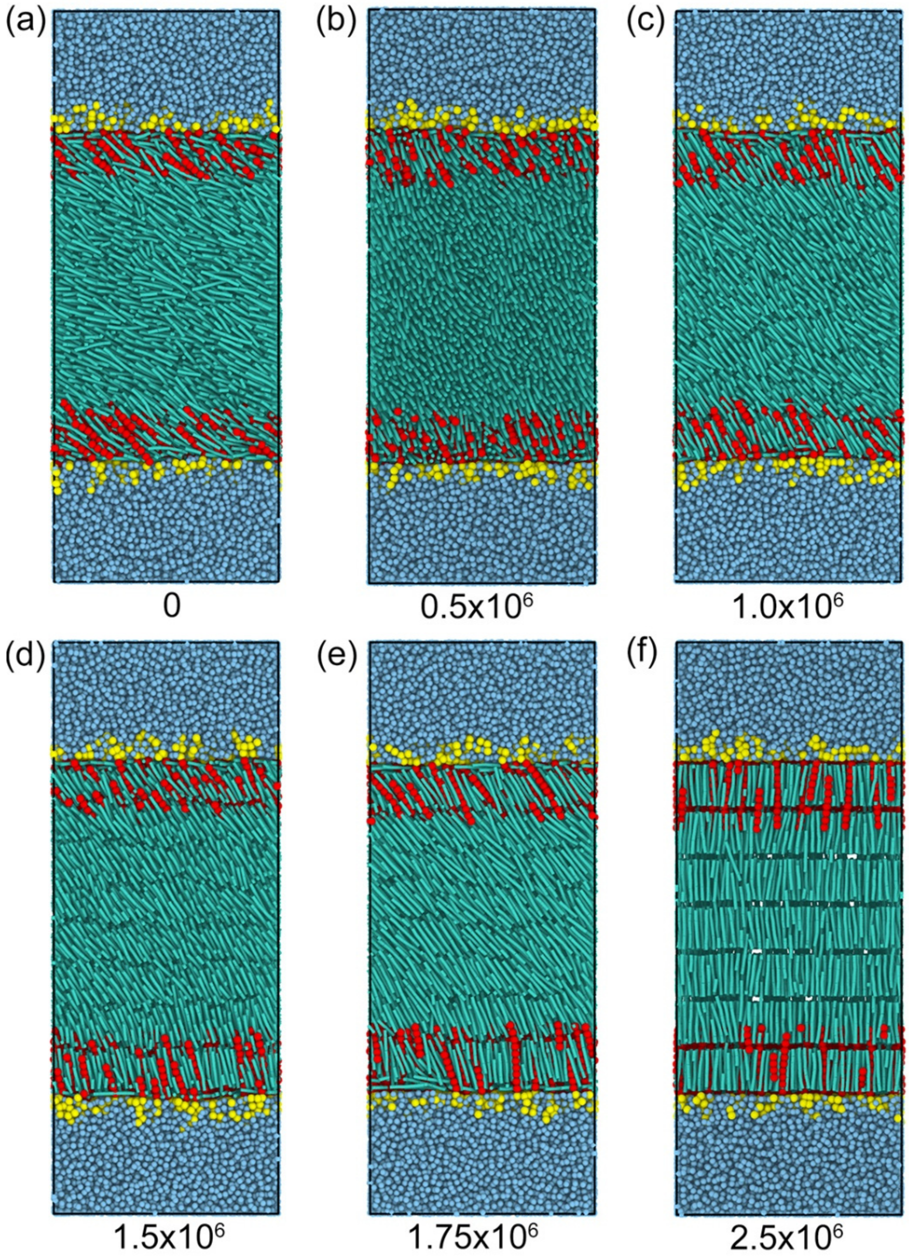

Figure S3. Instantaneous snapshots of six representative configurations in a reorientation process by cooling the tilted anchoring configuration at ρ_s = 0.4 and T = 0.6 to the homeotropic smectic anchoring at T = 0.3, when (a) t = 0, (b) t = 0.5×10^6 time steps, (c) t = 1.0×10^6 time steps, (d) t = 1.5×10^6 time steps, (e) t = 1.75×10^6 time steps, and (f) t = 2.5×10^6 time steps.
